# Quantitative HRCT-Derived Fibrosis Burden as an Independent Predictor of Mortality in Patients with Idiopathic Pulmonary Fibrosis: A Retrospective Observational Study

**DOI:** 10.3390/jcm15156117

**Published:** 2026-08-06

**Authors:** Burcu Akkok, Hatice Sahin, Betul Kizildag

**Affiliations:** 1 Department of Pulmonary Medicine, University of Health Sciences, Bakirkoy Dr. Sadi Konuk Training and Research Hospital, Zuhuratbaba, Dr. Tevfik Sağlam Cad. No. 11, Bakırköy, İstanbul 34147, Türkiye; 2 Department of Pulmonary Medicine, Faculty of Medicine, Kahramanmaraş Sütçü İmam University, Kahramanmaraş 46040, Türkiye; drh.sahin@hotmail.com; 3 Department of Radiology, Faculty of Medicine, Kahramanmaraş Sütçü İmam University, Kahramanmaraş 46040, Türkiye; drbetulkizildag@hotmail.com

**Keywords:** computed tomography, coronary artery disease, idiopathic pulmonary fibrosis, mortality, prognosis

## Abstract

**Objectives:** Idiopathic pulmonary fibrosis (IPF) is a progressive interstitial lung disease with increasing prevalence and mortality. High-resolution computed tomography (HRCT) is routinely performed in IPF assessment and can provide additional quantitative information. However, the prognostic utility of these HRCT-based parameters in IPF remains uncertain. This study aimed to investigate the prognostic value of quantitative HRCT findings in predicting mortality among patients with IPF. **Methods:** In this retrospective cohort study, 48 patients diagnosed with IPF between 2014 and 2024 were analyzed. Demographics, pulmonary function tests, HRCT findings, and quantitative measurements, including coronary artery calcium (CAC) score; densities of hepatic, paraspinal muscle, and lumbar vertebral bone mineral; and HRCT-derived fibrosis scores, were collected at baseline and after at least two years. The primary outcome was all-cause mortality. **Results:** The mean age was 66.8 ± 8.5 years; 77.1% were male. During a median follow-up of 63.3 months, 22 patients (45.8%) died, mainly from IPF-related causes (71.4%). Non-survivors had significantly higher HRCT fibrosis scores both at diagnosis (*p* = 0.006) and at two years (*p* = 0.002). Fibrosis scores increased significantly over time in non-survivors (*p* = 0.019). CAC scores rose in both groups, with a greater increase in non-survivors, but their independent prognostic value was limited after adjustment. Multivariable analysis identified male sex (hazard ratio [HR]: 5.46, *p* = 0.031) and second-year fibrosis score (HR: 1.10, *p* < 0.001) as independent predictors of mortality. **Conclusions:** HRCT-derived fibrosis burden is an independent predictor of mortality in IPF, alongside male sex. While other HRCT-based measures showed limited prognostic significance, longitudinal increases in CAC scores suggested potential cardiovascular implications.

## 1. Introduction

Idiopathic pulmonary fibrosis (IPF) is a chronic, progressive fibrosing interstitial pneumonia [1]. It is considered the prototypical restrictive lung disease, with a steadily increasing prevalence and mortality rate [2,3,4]. There is a strong relationship between IPF and coronary artery disease (CAD), which significantly contributes to the high mortality rates observed in IPF patients [2,5]. Furthermore, overlapping symptoms such as dyspnea and the exacerbation of ischemic events due to hypoxia or pulmonary hypertension (HT) may obscure the diagnosis of concurrent IPF and CAD [6]. Therefore, the coronary artery calcium (CAC) score, which reflects coronary atherosclerotic burden rather than a definitive diagnosis of obstructive CAD, may help predict mortality and adverse clinical events in IPF patients [3,5,7,8]. High-resolution computed tomography (HRCT) is routinely used to assess the extent and morphology of IPF [9,10]. Despite limitations such as inter-observer variability, respiratory motion artifacts, inconsistent inspiratory depth, and limited visual assessment due to time constraints, HRCT is gaining traction in clinical practice, especially when interpreted using qualitative and quantitative methods [10,11]. Semi-quantitative or automated quantitative scoring of HRCT findings has shown prognostic value during the longitudinal follow-up of IPF patients [4,11]. HRCT imaging has also been used to assess fatty infiltration in intra-abdominal solid organs and muscles through tissue density measurements in the context of various systemic inflammatory disorders [7,12,13].

Another parameter affected by chronic respiratory diseases is bone mineral density (BMD) [13]. Studies have reported significantly decreased BMD in patients with diffuse interstitial or chronic obstructive lung diseases [13,14]. Given the clinical similarities between IPF and these chronic lung diseases, it is also possible that IPF predisposes patients to bone loss. In light of this information, the objective of this study is to assess the prognostic value of HRCT-based parameters, including CAC score, hepatic density, paraspinal muscle density, and lumbar vertebral density, in predicting mortality in a well-characterized cohort of patients with IPF.

## 2. Materials and Methods

### 2.1. Study Design and Setting

This study was designed as a retrospective observational single-center cohort study and performed per the ethical considerations outlined in the Declaration of Helsinki. This study was approved by the Kahramanmaras Sütçü Imam University Medical Research Ethics Committee (protocol no: 2024/32-08; approval date: 2 December 2024). The requirement to obtain written informed consent was waived due to the retrospective design of the study and the anonymity of the data.

### 2.2. Population and Sample

**Figure 1:**
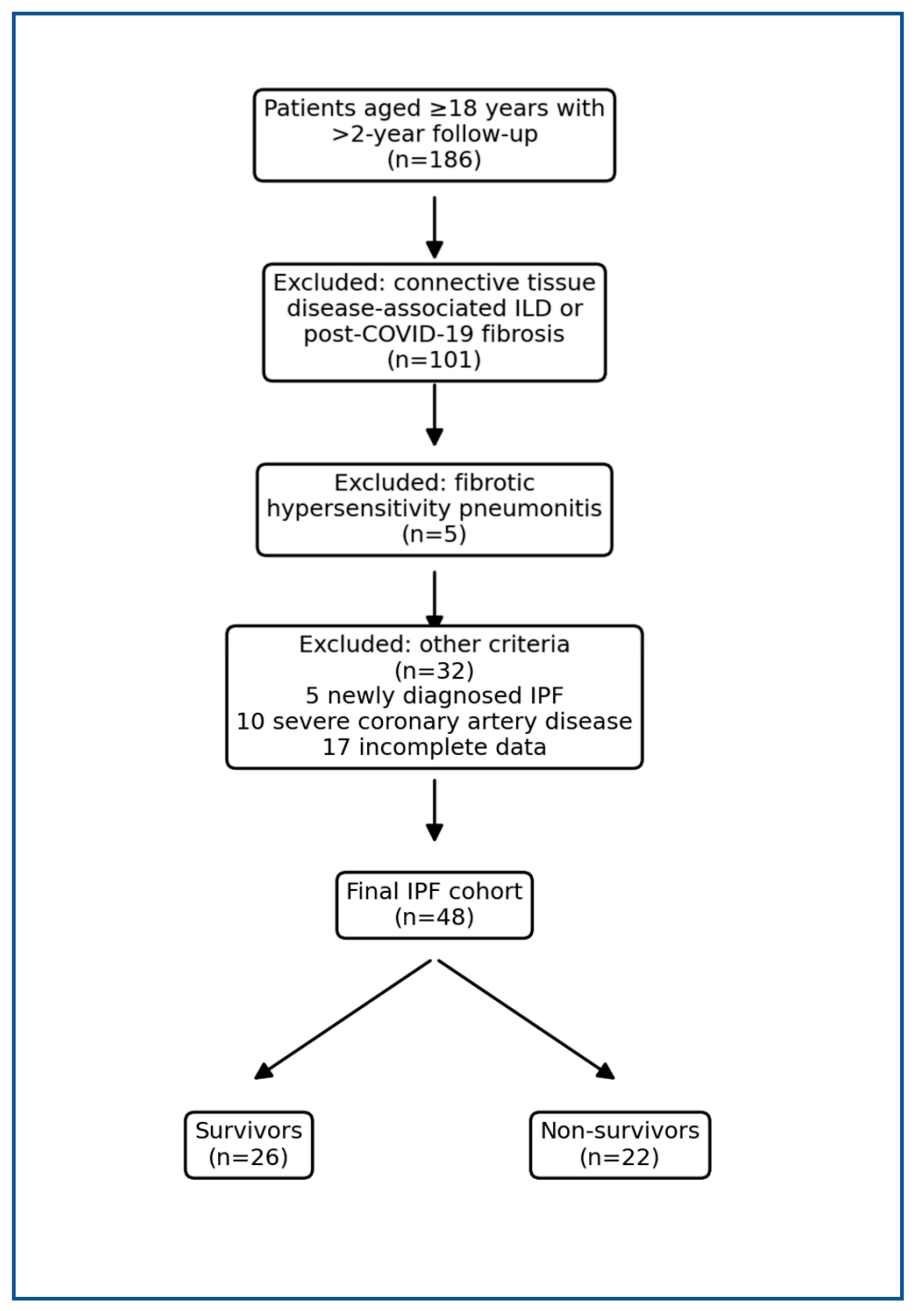
Flow diagram of patient selection and study process.

This study included consecutive IPF patients followed between January 2014 and December 2024. Diagnosis was based on usual interstitial pneumonia (UIP) or probable UIP pattern on HRCT per ATS/ERS/JRS/ALAT guidelines, supported by restrictive pulmonary function tests (forced vital capacity [FVC] < 80% predicted and/or forced expiratory volume in 1 s [FEV_1_]/FVC ≥ 0.70). IPF diagnoses were categorized as probable or suspicious based on imaging findings [15,16]. Of 186 patients aged ≥18 years with >2-year follow-up, exclusions included 101 with connective tissue disease-associated interstitial lung disease (ILD) or post-COVID-19 fibrosis, five with fibrotic hypersensitivity pneumonitis, and 32 with other exclusion criteria (5 in whom the scheduled second-year HRCT could not be obtained because patients did not attend the follow-up visit, precluding second-year assessment; 10 with severe coronary artery disease; and 17 with incomplete data). The final cohort comprised 48 IPF patients: survivors and non-survivors (Figure 1).

### 2.3. Data Collection

Patients’ demographic characteristics, smoking histories, comorbidities, antifibrotic treatment histories, pulmonary function test (PFT) results, and HRCT findings were collected and analyzed. The gender–age–physiology (GAP) index score was calculated for each patient as described previously [17]. Pulmonary function tests, including spirometry (FVC, FEV_1_, FEV_1_/FVC) and diffusing capacity of the lungs for carbon monoxide (DLCO), were performed within a few weeks of the HRCT scan using post-bronchodilator values. Testing followed American Thoracic Society/European Respiratory Society (ATS/ERS) technical and quality-control standards, and percent predicted values were calculated with the Global Lung Function Initiative (GLI) 2012 reference equations.

### 2.4. HRCT Acquisition Protocol

The HRCT scans were acquired without intravenous contrast in the supine position during full inspiration using a 320-detector row multidetector computed tomography (CT) scanner (Aquilion ONE ViSION Edition; Canon Medical Systems Corporation, Otawara, Japan), covering the entire lung from the apex to base in helical mode. Acquisition parameters were as follows: detector width: 320 × 0.5 mm; tube voltage: 120 kV; tube current: 280 mAs; slice thickness: 0.5 mm; slice interval: 1.5 mm; rotation time: 0.275 s; pitch factor: 1.388; and a variable field of view (FOV) between 35 and 45 cm (e.g., 40 × 40 cm). A 32 cm diameter phantom was used to simulate an average adult body. The average volume CT dose index (CTDIvol) and dose length product (DLP) were 4.8 mGy and 182.7 mGy·cm, respectively.

Quantitative image analysis was performed using the “Lung Density Analysis” software in a fully automated manner on a dedicated workstation (Vitrea; version 7.14.2.227 Canon Medical Systems Corporation, Otawara, Japan) by a radiologist (BK) with 25 years of experience in interstitial lung disease, blinded to clinical and functional data. HRCT fibrosis scores were calculated as defined previously [18].

### 2.5. Coronary Artery Calcium Score

The CAC score was determined for each patient on the left main, left anterior descending, circumflex, and right coronary arteries. The total CAC score was calculated by summing the scores of individual lesions using a commercial software (Vitrea, version 7.14.2.227, Vital Images Inc., Minnetonka, MN, USA) which were graded according to the Agatston method, i.e., 0 = no visible calcification, 1 = trace of calcification (Agatston score 1–10), 2 = mild calcification (Agatston score 11–100), 3 = moderate calcification (Agatston score 101–400), and 4 = severe calcification (Agatston score > 400) [3,19]. Calcifications were automatically identified based on lesions with a peak attenuation greater than 130 Hounsfield units (HU).

### 2.6. Hepatic Attenuation

Hepatic attenuation was measured by averaging HU values from 1.5 cm^2^ circular regions of interest (ROIs). Two ROIs were placed at two different sites within each of the right hepatic lobe segments (segments V, VI, VII, and VIII as defined by the Couinaud classification) [20].

### 2.7. Skeletal Muscle Radiation Attenuation Index

Paraspinal muscle measurements were conducted to calculate the skeletal muscle radiation attenuation index (HU), primarily at the Th12–L1 level, with L3–L4 used when values at Th12–L1 could not be reliably obtained [12].

### 2.8. Lumbar Vertebral Bone Density

L1 vertebral bone density was measured using axial, sagittal, and coronal HRCT images. The measurement area was selected to be as large as possible, excluding the cortical bone, as previously described [21]. A commercial software (Vitrea, version 7.14.2.227, Vital Images Inc., Minnetonka, MN, USA) was used to determine the HU values of each plane. The average of the measurements performed by the same physician for all patients in HU was regarded as the mean L1 vertebral bone density.

### 2.9. Follow-Up Procedure

Due to the data collection process, follow-up duration and overall survival (OS) were calculated separately. Follow-up duration spanned from diagnosis (or treatment initiation) to the last clinical contact, regardless of patient status. OS was defined as time from diagnosis to death from any cause; for patients alive or lost to follow-up, the last contact date was used.

Given the retrospective design, some patients’ recorded follow-up durations were shorter than their actual survival times, as their deaths predated data collection. This discrepancy was accounted for in survival analyses.

A second HRCT was performed ≥2 years after initial IPF diagnosis to assess longitudinal changes in: (1) coarseness score and ILD grade (fibrosis progression); (2) CAC scores (vascular calcification); (3) hepatic attenuation (metabolic status); (4) paraspinal muscle area and density (muscle mass/quality); and (5) lumbar vertebral BMD (skeletal changes). All measurements used the same software, workstation, and standardized protocol.

### 2.10. Statistical Analysis

The impact of HRCT-derived parameters on prognosis was analyzed over a follow-up period of at least two years from diagnosis. The primary outcome was mortality; secondary outcomes were differences in HRCT-derived parameters between survivors and non-survivors during follow-up.

Statistical analyses were performed using Jamovi 2.6.44 and JASP 0.19.3. Results were expressed as mean ± standard deviation (normally distributed continuous variables), median with minimum–maximum (non-normally distributed variables), and frequencies/percentages (categorical variables). Normality was assessed using the Shapiro–Wilk test. For continuous variables, the independent samples *t*-test (normal distribution) or Mann–Whitney U test (non-normal distribution) were used between two groups; for categorical variables, Fisher’s exact test (expected cell count < 5) or the chi-square test were applied. Within-group longitudinal comparisons used the paired *t*-test or Wilcoxon signed-rank test, as appropriate. Percentage changes between baseline and two-year measurements were compared between groups using the Mann–Whitney U test. Bonferroni correction was applied to multiple comparisons, with adjusted significance set at α = 0.0028 (0.05/18 comparisons).

Overall survival (OS), rather than follow-up duration, was used as the time variable in all survival analyses. Survival analyses used the Kaplan–Meier method, with log-rank tests for inter-group comparisons; three- and five-year survival rates were calculated. Cox proportional hazards regression identified mortality predictors. Variables significant at *p* < 0.25 in univariable analysis were entered into four multivariable models; Model 4 used backward stepwise elimination (entry *p* < 0.05, removal *p* > 0.10). The proportional hazards assumption was verified via Schoenfeld residuals. Model performance was evaluated using the concordance index and likelihood-ratio test. Hazard ratios (HRs) were reported with 95% confidence intervals. All tests were two-tailed, with *p* ≤ 0.05 considered significant. Analyses used available data only; no imputation was performed for missing data. No a priori sample size calculation was performed; given the retrospective design, the cohort size was determined by the number of eligible patients over the study period rather than by a pre-specified power calculation, and the reported effect sizes and confidence intervals should therefore be interpreted as exploratory rather than confirmatory. Variance inflation factors (VIFs) and pairwise correlations were also calculated to assess collinearity between FVC and FEV_1_ in Model 1, and a sensitivity analysis excluding FEV_1_ was performed.

## 3. Results

The mean age of the sample was 66.8 ± 8.5 years. There was no significant difference between the survivor and non-survivor groups in age (*p* = 0.310). There were 37 (77.1%) males and 11 (22.9%) females in the sample. The rate of males was higher, but not significantly, in the non-survivor group than in the survivor group (90.9% and 65.4%, respectively, *p* = 0.08).

HT was present in 23 (47.9%) patients. There was no significant difference between the study groups in other baseline demographic and clinical characteristics (*p* > 0.05) (Table 1).

The median disease duration of the sample was 60 months. There was no significant difference between the groups in median disease duration (*p* = 0.48). Most (93.8%) patients were on antifibrotic therapy. There was also no significant difference between the groups in the rate of patients receiving antifibrotic therapy (*p* = 0.59) (Table 2).

The PFT results of the non-survivor group were significantly worse than those of the survivor group. Specifically, FVC (% predicted), FEV_1_ (% predicted), and the diffusing capacity of the lungs for carbon monoxide (DLCO) (% predicted) were all significantly lower in the non-survivor group than in the survivor group (*p* < 0.05 for all). There was no significant difference between the groups in FEV_1_/FVC ratio (*p* = 0.510).

Reticulation (91.7%), honeycomb appearance (77.1%), fibrosis (56.2%), and ground-glass opacities (56.2%) were the most frequent imaging findings seen in the sample, with no significant difference between the groups in any of these (*p* > 0.05) (Table 2).

There were no significant differences in baseline and two-year GAP index scores between the groups (*p* > 0.05). On the other hand, the median baseline and two-year HRCT fibrosis scores of the non-survivor group were significantly higher than those of the survivor group (25.3 vs. 16.7, *p* = 0.006, and 28.5 vs. 16.4, *p* = 0.002). The significant difference between the groups in median two-year HRCT fibrosis score was retained after Bonferroni correction (adjusted α = 0.0028) (Table 3).

HRCT fibrosis scores significantly increased over time in the non-survivor group (*p* = 0.019), but not in the survivor group (*p* = 0.298). The median two-year CAC score of the non-survivor group was significantly higher than that of the survivor group (32.5 vs. 3.5, *p* = 0.005), yet this difference between groups lost significance after Bonferroni correction. CAC scores increased significantly over the follow-up period in both groups, with the increase in the non-survivor group (*p*< 0.001) remaining statistically significant after Bonferroni correction. Other radiographic parameters, including lumbar vertebra density and vertebral muscle density, changed significantly in the survivor and non-survivor groups, respectively (*p* = 0.017 and *p* = 0.033), yet these changes lost significance after adjustment for multiple testing. There were no significant differences in other quantitative measurements and their longitudinal changes (*p* > 0.05) (Table 3).

The median follow-up period of the patients was 63.3 (min. 7.4, max. 142.4) months. The median follow-up period of the survivor group was significantly longer than that of the non-survivor group (70.6 vs. 44.6 months, *p* = 0.035). Hospitalization was required in 35.4% of the patients. IPF was the leading cause of admission in nine of the 11 hospitalized patients in the non-survivor group.

The median OS was 5.0 (min 0.5, max 12) years. The median OS of the non-survivor group was shorter, albeit not significantly, than that of the survivor group (3.2 years vs. 5.0 years, *p* = 0.083). The 3- and 5-year survival rates were significantly higher in the survivor group than in the non-survivor group (*p* = 0.003 and *p* < 0.001). The primary cause of death in 22 (45.8%) of the 48 patients who died was IPF-related causes, which were recorded as the cause of death in 15 (71.4%) of the deceased patients (Table 4). The incidence of major adverse cardiovascular events (MACEs) in the sample was 18.8%, with no significant difference in rates of patients with MACEs or various MACE types between the groups (*p* > 0.05) (Table 4).

**Figure 2:**
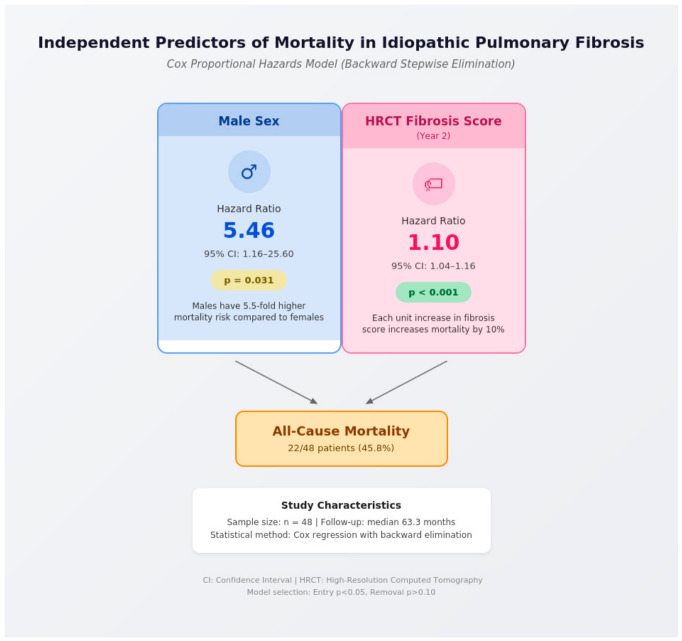
Independent predictors of all-cause mortality in patients with idiopathic pulmonary fibrosis identified by Cox proportional hazards regression analysis. The Cox regression model with backward stepwise elimination (entry *p* < 0.05, removal *p* > 0.10) identified two independent predictors of mortality in IPF patients. Male sex (**left** panel, blue) was associated with a 5.46-fold increased mortality risk (95% CI: 1.16–25.60, *p* = 0.031), indicating that males have 5.5-fold higher mortality risk compared to females. The second-year HRCT fibrosis score (**right** panel, pink) demonstrated a hazard ratio of 1.10 (95% CI: 1.04–1.16, *p* < 0.001), signifying that each unit increase in fibrosis score increased mortality risk by 10%. The central panel shows the primary outcome of all-cause mortality, which occurred in 22 of 48 patients (45.8%) during a median follow-up period of 63.3 months. The arrows indicate the directional relationship between the predictors and the outcome. This final model was derived from a cohort of 48 IPF patients using Cox regression with the backward elimination methodology.

Cox regression analysis was used to identify the variables that significantly predict mortality. Accordingly, univariable analysis revealed that pulmonary function parameters, specifically FVC (HR: 0.97, 95% CI: 0.94–1.00, *p* = 0.023) and FEV_1_ (HR: 0.97, 95% CI: 0.94–1.00, *p* = 0.04), were significantly associated with reduced mortality risk. The two-year HRCT fibrosis score also significantly predicted all-cause mortality in IPF patients (HR: 1.07, 95% CI: 1.03–1.12, *p* = 0.001). The multivariable model with backward elimination (Model 4) revealed male gender (HR: 5.46, 95% CI: 1.16–25.60, *p* = 0.031) and two-year HRCT fibrosis score (HR: 1.10, 95% CI: 1.04–1.16, *p* < 0.001) as independent predictors of mortality (Table 5, Figure 2). Variance inflation factors for FVC and FEV_1_ in Model 1 were both 2.93, below the threshold generally taken to indicate problematic collinearity (VIF > 5), despite a moderate correlation between the two variables (r = 0.81). Excluding FEV_1_ from the candidate variables in a sensitivity analysis left the final model estimates essentially unchanged, indicating that this collinearity did not affect the study’s main conclusions.

## 4. Discussion

This study highlights the prognostic value of quantitative imaging markers in IPF. Baseline and two-year HRCT fibrosis scores were significantly higher in non-survivors and increased over time in both groups, supporting regular fibrosis quantification for mortality risk stratification. Two-year CAC scores were also higher in non-survivors, though this difference lost significance after multiple comparison correction; the longitudinal CAC increase nonetheless suggests a link between subclinical cardiovascular burden and adverse outcomes. Other radiographic indices (vertebral muscle and bone density) similarly lost significance after correction, indicating limited prognostic utility. Overall, incorporating dynamic HRCT-derived fibrosis metrics into routine assessment may improve risk stratification and guide management in IPF.

The relationship between HRCT findings and clinical outcomes in IPF has been studied previously [1,4,10]. Although several methods assess pulmonary function, disease progression, and mortality, no widely accepted HRCT-based method reliably correlates with pulmonary function or predicts mortality [4]. Rea et al. [22] and Torrisi et al. [11] found that quantitative HRCT parameters, such as mean lung density and fibrotic/high-attenuation areas, served as proxy markers of disease severity and survival, though methodological variations and non-standardized reporting limit comparisons across studies [10]. Lee et al. [9] reported reduced survival with increased fibrosis, reticulation, honeycombing, consolidation, and emphysema scores, with reticulation having the greatest impact, while preserved FVC/DLCO improved survival. In contrast, we found no difference in the qualitative HRCT findings (reticulation, honeycombing, ground-glass opacity) between survivors and non-survivors, though we did not assess these parameters separately by severity; notably, our quantitative HRCT fibrosis score—unlike these categorical findings—did differ significantly between groups, consistent with its role as an independent mortality predictor.

IPF is more common in males. While most studies reported male gender as a negative prognostic factor associated with mortality in IPF patients, in line with this finding, others have reported findings to the contrary [8,9]. In comparison, the male-to-female ratio in our sample was 3.36, and we found male gender and two-year HRCT fibrosis score to be independent risk factors for mortality. The potential relationship between male gender and mortality in IPF patients needs to be elucidated by future causal analyses that account for additional confounding factors such as pulmonary function parameters and HRCT findings.

The relationship between cardiovascular disease and mortality in IPF has been well-documented. Caminati et al. [8] found that male gender and higher baseline CAC score predicted mortality. Sinha et al. [23] reported higher CAD rates among IPF-related hospitalizations. Higher CAC scores have been associated with a greater burden of underlying coronary atherosclerosis in IPF [2,3]. A CHA2DS2-VASc score > 4 identifies high mortality and rehospitalization risk, and is reportedly more effective than CAC score [5]. We did not assess CHA2DS2-VASc utility. We found significantly higher follow-up CAC scores in non-survivors, yet multivariate analysis showed no significant relationship with mortality. IPF-related conditions occurred more frequently than MACEs.

In IPF patients, various muscles such as the erector spinae and pectoralis have been assessed using CT-based tools including skeletal muscle area, mass index, and radiation attenuation index [12,24,25,26,27,28]. Skeletal muscle radiation attenuation index is a quantitative parameter reflecting muscle tissue quality through indirect analysis of intramuscular fat deposition. Although L3 is typically used as the cut-off level for skeletal muscle measurements, L1 is cited as another viable alternative [12]. Accordingly, we assessed skeletal muscle radiation attenuation index at two different levels. Nakano et al. [24] reported that reduced erector spinae muscle, likely due to sarcopenia and cachexia, is a significant prognostic factor for mortality in IPF. Cinkooglu et al. [26] found that erector spinae muscle area predicted short- and long-term survival in IPF. These studies interpreted reduced erector spinae area as indicating sarcopenia, despite contradictory pectoralis findings [24,26,27]. Small muscle mass may be insufficient to reflect whole-body muscle mass [26,28,29]. Moon et al. [27] found that low skeletal mass index (pectoralis, paraspinal, serratus, latissimus) normalized for stature may strongly predict all-cause mortality in IPF. In contrast, we found no significant relationship between paraspinal muscle density at Th12–L1 and L3–L4 levels and mortality.

Muscle density can be used to assess the relationship between muscle quality and mortality in chronic disease patients, with contradictory results [26,30]. However, differences in patient/disease characteristics and muscle anatomy/imaging features may explain varying outcomes. HRCT-assessed thoracic vertebral BMD is another parameter used to evaluate IPF prognosis. Ikezoe et al. [13] reported significantly lower thoracic vertebral BMD in IPF patients, with a negative correlation between BMD and emphysema extent. In contrast, we found no significant relationship between L1 vertebral bone density and mortality.

Serial HRCT assessment could improve risk stratification, but applying it routinely is not straightforward. Repeated scans add radiation exposure and cost, reimbursement is inconsistent, and access to imaging varies across healthcare settings. A more realistic approach may be to apply quantitative fibrosis scoring to HRCT scans already obtained for clinical reasons, such as disease monitoring, rather than scheduling additional scans purely for risk stratification.

This study has limitations. First, its retrospective, single-center design may limit generalizability to broader IPF populations. Second, the small sample size (*n* = 48) and limited number of events (22 deaths) reduced statistical power, widened the confidence intervals around hazard ratio estimates, and limited how many covariates could be reliably included in the multivariable models; these results should be considered hypothesis-generating and need confirmation in larger, multicenter cohorts. Third, differences in imaging acquisition and reconstruction across studies may limit comparability, despite internal standardization. Fourth, we did not assess markers like the CHA_2_DS_2_-VASc score or comorbidity burden. Fifth, the lack of separate reticulation/honeycombing scoring may have obscured feature-specific associations. Sixth, CAC scoring was performed on non-ECG-gated HRCT rather than a dedicated cardiac CT protocol; although CAC can be reliably estimated on non-gated chest CT, motion artifacts, slice thickness, and acquisition parameters may reduce its precision compared with gated acquisitions. Seventh, only percent-predicted spirometric values were retained in our database; the raw absolute spirometric volumes and the specific reference equations used at the time of testing were not documented, precluding reliable retrospective calculation of Global Lung Function Initiative-based z-scores, which are less affected by age, sex, and height. Finally, the observational design precludes causal inference. Additionally, the use of the second-year HRCT fibrosis score as a predictor of mortality is subject to immortal time bias, as only patients who survived to the two-year assessment could contribute this value; consequently, the observed association may partly reflect survivorship rather than a purely causal predictive relationship. Future studies should consider landmark analyses or time-varying covariate modeling to address this limitation.

## 5. Conclusions

In conclusion, two-year HRCT-derived fibrosis score and male gender emerged as independent predictors of mortality in IPF. Although CAC score, vertebral muscle density, and BMD showed limited prognostic value after adjustment, the consistent CAC increase over time suggests a link between subclinical cardiovascular disease and adverse outcomes. These findings support incorporating quantitative HRCT fibrosis assessment into follow-up, whether on a scheduled basis or on clinically indicated scans, for earlier risk identification and personalized management. Future studies should validate standardized fibrosis scoring, integrate imaging biomarkers with functional and clinical parameters, and explore targeted interventions for high-risk patients.

## Figures and Tables

**Table 1:** Demographic and clinical characteristics of IPF patients stratified by survival status.

	Overall (*n* = 48)	No Survivors (*n* = 22)	Survivors (*n* = 26)	*p*
Age (years)	66.8 ± 8.5	65.4 ± 9.7	68.0 ± 7.3	0.310
Sex				
Female	11 (22.9)	2 (9.1)	9 (34.6)	0.080
Male	37 (77.1)	20 (90.9)	17 (65.4)	
Body mass index (kg/m^2^)	26.5 ± 4.4	27.1 ± 4.2	26.0 ± 4.5	0.381
Smoking				
Non-smoker	24 (50.0)	10 (45.5)	14 (53.8)	0.871
Smoker	8 (16.7)	4 (18.2)	4 (15.4)	
Ex-smoker	16 (33.3)	8 (36.4)	8 (30.8)	
Cigarette consumption (pack/year)	30.0 [20.0–40.0]	25.0 [20.0–35.0]	30.0 [20.0–40.0]	0.649
Coexisting diseases	25 (52.1)	9 (40.9)	16 (61.5)	0.256
Hypertension	23 (47.9)	7 (31.8)	16 (61.5)	0.078
Diabetes mellitus	8 (16.7)	4 (18.2)	4 (15.4)	0.999
Hyperlipidemia	3 (6.2)	0 (0.0)	3 (11.5)	0.239
Others	2 (4.2)	0 (0.0)	2 (7.7)	0.493

Values are presented as mean ± standard deviation, median [minimum–maximum], or *n* (%). IPF: idiopathic pulmonary fibrosis.

**Table 2:** Disease-related characteristics of IPF patients according to survival status.

	Overall (*n* = 48)	No Survivors (*n* = 22)	Survivors (*n* = 26)	*p*
Disease duration (months)	60.0 [7.0–160.0]	58.0 [7.0–160.0]	65.0 [12.0–120.0]	0.480
Medications				
Steroids	12 (25.0)	8 (36.4)	4 (15.4)	0.181
Immunosuppressants	3 (6.2)	2 (9.1)	1 (3.8)	0.587
Antifibrotics	45 (93.8)	20 (90.9)	25 (96.2)	0.587
Type of antifibrotics				
Nintedanib	19 (43.2)	8 (40.0)	11 (45.8)	0.934
Pirfenidon	25 (56.8)	12 (60.0)	13 (54.2)	
Diagnosis groups based on CT findings				
Probable	27 (56.2)	13 (59.1)	14 (53.8)	0.942
Suspicious	21 (43.8)	9 (40.9)	12 (46.2)	
Respiratory function tests				
FVC (% predicted)	77.2 ± 18.2	70.0 ± 19.6	83.3 ± 14.8	0.012
FEV_1_ (% predicted)	81.2 ± 15.1	75.1 ± 15.8	86.5 ± 12.5	0.010
DLCO (% predicted)	60.4 ± 12.6	56.2 ± 11.4	63.8 ± 12.7	0.036
FEV_1_/FVC	104.6 ± 15.8	106.3 ± 15.6	103.2 ± 16.1	0.510
Computed tomography findings				
Fibrosis	27 (56.2)	12 (54.5)	15 (57.7)	0.999
Reticulation	44 (91.7)	22 (100.0)	22 (84.6)	0.114
Emphysema	6 (12.5)	2 (9.1)	4 (15.4)	0.674
Honeycomb appearance	37 (77.1)	18 (81.8)	19 (73.1)	0.709
Ground-glass opacity	27 (56.2)	13 (59.1)	14 (53.8)	0.942

Values are presented as mean ± standard deviation, median [minimum–maximum], or *n* (%). IPF: idiopathic pulmonary fibrosis; CT: computed tomography; FVC: forced vital capacity; FEV_1_: forced expiratory volume in 1 s; DLCO: diffusing capacity for carbon monoxide.

**Table 3:** Longitudinal assessment of radiographic parameters in IPF patients stratified by survival status.

Parameters		No Survivors (*n* = 22)	Survivors (*n* = 26)	*p*
GAP index	Baseline	3.5 [1.0–8.0]	3.0 [0.0–6.0]	0.193
	2nd year	3.0 [1.0–8.0]	3.0 [0.0–5.0]	0.407
*p*		0.931	0.097	
HRCT fibrosis score	Baseline	25.3 [12.8–53.7]	16.7 [8.9–44.7]	0.006
	2nd year	28.5 [9.5–57.9]	16.4 [9.2–44.9]	0.002
*p*		0.019	0.298	
Coronary artery calcium score	Baseline	0.0 [0.0–900.0]	0.0 [0.0–23.0]	0.105
	2nd year	32.5 [0.0–1050.0]	3.5 [0.0–76.0]	0.005
*p*		<0.001	0.002	
Hepatic density (HU)	Baseline	56.7 ± 9.4	58.1 ± 6.8	0.562
	2nd year	58.7 ± 10.5	58.2 ± 7.5	0.830
*p*		0.297	0.681	
Paraspinal muscle density (HU)	Baseline	43.1 [31.0–53.0]	44.8 [28.6–74.9]	0.228
	2nd year	47.7 [31.8–82.7]	48.6 [21.0–122.5]	0.966
*p*		0.033	0.937	
Lumbar vertebral density (HU)	Baseline	104.4 [72.8–198.7]	106.0 [52.0–206.4]	0.893
	2nd year	101.9 [78.3–204.0]	100.3 [40.1–159.5]	0.792
*p*		0.291	0.017	

Values are presented as mean ± standard deviation or median [minimum–maximum]. IPF: idiopathic pulmonary fibrosis; GAP: gender, age, and physiology; HRCT: high-resolution computed tomography; HU: Hounsfield units.

**Table 4:** Clinical outcomes and survival analysis of IPF patients.

	Overall (*n* = 48)	No Survivors (*n* = 22)	Survivors (*n* = 26)	*p*
Follow-up (months)	63.3 [7.4–142.4]	44.6 [7.4–129.2]	70.6 [17.7–142.4]	0.035
Need for hospitalization	17 (35.4)	11 (50.0)	6 (23.1)	0.101
Reason for hospitalization				
Cardiac pathologies	5 (29.4)	0	5	
IPF	10 (58.8)	9	1	
Others	2 (11.8)	1	1	
Overall survival (years)	5.0 [0.5–12.0]	3.2 [0.5–11.0]	5.0 [1.0–12.0]	0.083
3-year survival	40 (85.1)	15 (68.2)	25 (100.0)	0.003
5-year survival	27 (67.5)	9 (40.9)	18 (100.0)	<0.001
Reason for mortality				
IPF-related	-	15 (71.4)	-	-
MACE	-	1 (4.8)	-	
Others (COVID-19 infection, earthquake, sepsis, pulmonary embolism)	-	5 (23.8)	-	
MACE	9 (18.8)	4 (18.2)	5 (19.2)	0.999
Type of MACE				
Myocardial infarction	5 (10.4)	3 (13.6)	2 (7.7)	0.649
Ischemic heart disease	7 (14.6)	3 (13.6)	4 (15.4)	0.999
Heart failure	2 (4.2)	1 (4.5)	1 (3.8)	0.999

Values are presented as median [minimum–maximum] or *n* (%). IPF: idiopathic pulmonary fibrosis; MACE: major adverse cardiovascular event.

**Table 5:** Univariable and multivariable Cox regression analyses of factors predicting mortality in patients with idiopathic pulmonary fibrosis.

Variables	Univariable Analysis	Multivariable Model 1	Multivariable Model 2	Multivariable Model 3	Multivariable Model 4
	HR (95% CI)	HR (95% CI)	HR (95% CI)	HR (95% CI)	HR (95% CI)
Demographic Characteristics					
Age (years)	1.02 (0.96–1.07), *p* = 0.513	-	-	-	-
Sex (male)	3.74 (0.86–16.17), *p* = 0.078	3.42 (0.79–14.91), *p* = 0.101	-	-	5.46 (1.16–25.60), *p* = 0.031
Clinical Characteristics					
Hypertension, present	0.53 (0.20–1.38), *p* = 0.194	-	0.67 (0.27–1.68), *p* = 0.399	-	-
Pulmonary Function Tests					
FVC (% predicted)	0.97 (0.94–1.00), *p* = 0.023	0.97 (0.91–1.02), *p* = 0.237	-	-	-
FEV_1_ (% predicted)	0.97 (0.94–1.00), *p* = 0.040	1.01 (0.94–1.08), *p* = 0.758	-	-	-
DLCO (% predicted)	0.96 (0.92–1.00), *p* = 0.063	0.98 (0.94–1.03), *p* = 0.520	-	-	-
Radiological Parameters					
HRCT fibrosis score (baseline)	1.03 (0.99–1.07), *p* = 0.124	-	-	0.97 (0.93–1.02), *p* = 0.271	-
HRCT fibrosis score (2nd year)	1.07 (1.03–1.12), *p* = 0.001	-	1.07 (1.03–1.11), *p* = 0.001	-	1.10 (1.04–1.16), *p* < 0.001
CAC score (baseline)	1.00 (1.00–1.00), *p* = 0.595	-	-	-	-
CAC score (2nd year)	1.01 (0.99–1.01), *p* = 0.460	-	-	0.98 (0.96–1.00), *p* = 0.082	-

HR: hazard ratio; CI: confidence interval; FVC: forced vital capacity; FEV_1_: forced expiratory volume in one second; DLCO: diffusing capacity for carbon monoxide; HRCT: high-resolution computed tomography; CAC: coronary artery calcification. Model descriptions: Model 1: Basic demographic and functional model (sex + pulmonary function tests). Model 2: Radiological progression model (hypertension + 2nd year HRCT fibrosis score). Model 3: Systemic assessment model (baseline HRCT fibrosis score + 2nd year CAC score). Model 4: Final model obtained by backward elimination method (sex + 2nd year HRCT fibrosis score).

## Data Availability

The data that support the findings of this study are available from the corresponding author upon reasonable request, subject to institutional review board approval and appropriate data sharing agreements.
